# Multispecies fish tracking across newly created shallow and deep habitats in a forward-restored lake

**DOI:** 10.1186/s40462-023-00405-1

**Published:** 2023-07-27

**Authors:** Casper H. A. van Leeuwen, Joep J. de Leeuw, Olvin A. van Keeken, Joey J. J. Volwater, Ferdi Seljee, Roland van Aalderen, Willie A. M. van Emmerik, Elisabeth S. Bakker

**Affiliations:** 1 Department of Aquatic Ecology, Netherlands Institute of Ecology (NIOO-KNAW), Droevendaalsesteeg 10, 6708 PB Wageningen, The Netherlands; 2 Wageningen Marine Research, Wageningen UR, Haringkade 1, 1976 CP IJmuiden, The Netherlands; 3 Conservation Ecology Group, Groningen Institute of Evolutionary Life Sciences, Groningen University, Nijenborg 7, 9747 AG Groningen, The Netherlands; 4 Royal Dutch Angling Association, Leyenseweg 115, 3721 BC Bilthoven, The Netherlands; 5 Wildlife Ecology and Conservation Group, Wageningen UR, Droevendaalsesteeg 2, 6708 PB Wageningen, The Netherlands

**Keywords:** Acoustic telemetry, Deep sand excavation, Fish nursery, Macro-invertebrates, Marker Wadden, Forward-looking restoration, Shallow lake, Shelter

## Abstract

**Background:**

Freshwater fish communities typically thrive in heterogenous ecosystems that offer various abiotic conditions. However, human impact increasingly leads to loss of this natural heterogeneity and its associated rich fish communities. To reverse this trend, we need guidelines on how to effectively restore or recreate habitats for multiple fish species. Lake Markermeer in the Netherlands is a human-created 70,000-ha lake with a uniform 4 m-water depth, steep shorelines, high wind-induced turbidity, and a declining fish community. In 2016, a forward-looking restoration project newly created a 1000-ha five-island archipelago in this degrading lake, which offered new sheltered shallow waters and deep sand excavations to the fish community.

**Methods:**

In 2020, we assessed how omnivorous and piscivorous fish species used these new habitats by tracking 78 adult fish of five key species across local and lake-scales. We monitored spring arrival of adult fish and assessed local macro-invertebrate and young-of-the-year fish densities.

**Results:**

Adult omnivorous Cyprinidae and piscivorous Percidae arrived at the archipelago in early spring, corresponding with expected spawning movements. During the productive summer season, 12 species of young-of-the-year fish appeared along the sheltered shorelines, with particularly high densities of common roach (*Rutilus rutilus*) and European perch (*Perca fluviatilis*). This suggests the sheltered, shallow, vegetated waters formed new suitable spawning and recruitment habitat for the fish community. Despite highest food densities for adult fish in the shallowest habitats (< 2-m), adult fish preferred minimally 2-m deep water. After spawning most Cyprinidae left the archipelago and moved long distances through the lake system, while most Percidae remained resident. This may be related to (1) high densities of young-of-the-year fish as food for piscivores, (2) medium food densities for omnivores compared to elsewhere in the lake-system, or (3) the attractiveness of 30-m deep sand excavations that were newly created and frequently used by one-third of all tracked fish.

**Conclusions:**

New littoral zones and a deep sand excavation constructed in a uniform shallow lake that lacked these habitat types attracted omnivorous and piscivorous fish species within four years. Both feeding guilds used the littoral zones for reproduction and nursery, and notably piscivorous fish became residents year-round.

**Supplementary Information:**

The online version contains supplementary material available at 10.1186/s40462-023-00405-1.

## Introduction

Human developments greatly impact the world’s freshwater ecosystems [17, 48, 66], with human influence present in almost all lakes and rivers [25]. Common modifications that humans apply include fragmentation by dams, regulation of water levels, alteration of shoreline types, or combinations of these modifications in heavily impacted systems. A common denominator of human impact is that it often reduces the abiotic variation that is naturally present in ecosystems, either within ecosystems (e.g. all shorelines of a lake become more similar) or between ecosystems (e.g. all shorelines of all lakes look alike)—resulting in loss of ecological integrity from ecosystems [3, 48, 63]. Freshwater fish are important for the functioning of freshwater ecosystems, but among the globally most threatened organisms on the planet [83] and affected by all of the beforementioned human modifications [66]. Many fish species require different habitat types throughout their annual and life cycles, and therefore suffer from local habitat changes or barriers that prohibit movement between contrasting areas [11, 45, 74].

An important environmental variable for fishes is the water depth. In lakes, many fish species use both deeper waters and shallower sheltered, near-shore ecotones (“littoral zones” sensu [80]) throughout their lifetimes. Littoral zones typically offer feeding and spawning habitat with warmer, safer and more productive conditions for early life stages [5, 41, 56, 81]. Access to both shallower and deeper waters allows fish to select suitable habitat throughout their annual and life cycles. However, because also human modifications tend to concentrate along productive shorelines [57, 65, 76], worldwide many littoral and riparian zones in lakes and rivers are replaced by steeper basalt or asphalt dikes and dams for water safety—with cascading effects on fish communities.

Restoration measures that create more variation in water depths via engineering can be an effective approach to mitigate this human impact on fish, especially in waterbodies surrounded by barriers that prohibit fish movement. For instance, construction of shallow waters with gradually sloped shorelines in rivers increased fish recruitment in North-America [20]. Similarly, creation of shallow littoral zones in small temperate lakes in Europe had the potential to increase densities of young common roach (*Rutilus rutilus*) and European perch (*Perca fluviatilis*) [54]. However, it remains difficult to predict how fish species may start making use of newly created shallower or deeper areas in aquatic ecosystems that initially lacked such water depth variation, especially with regards to expected variation in preferences among species, feeding guilds and ontogenetic stages.

**Fig. 1:**
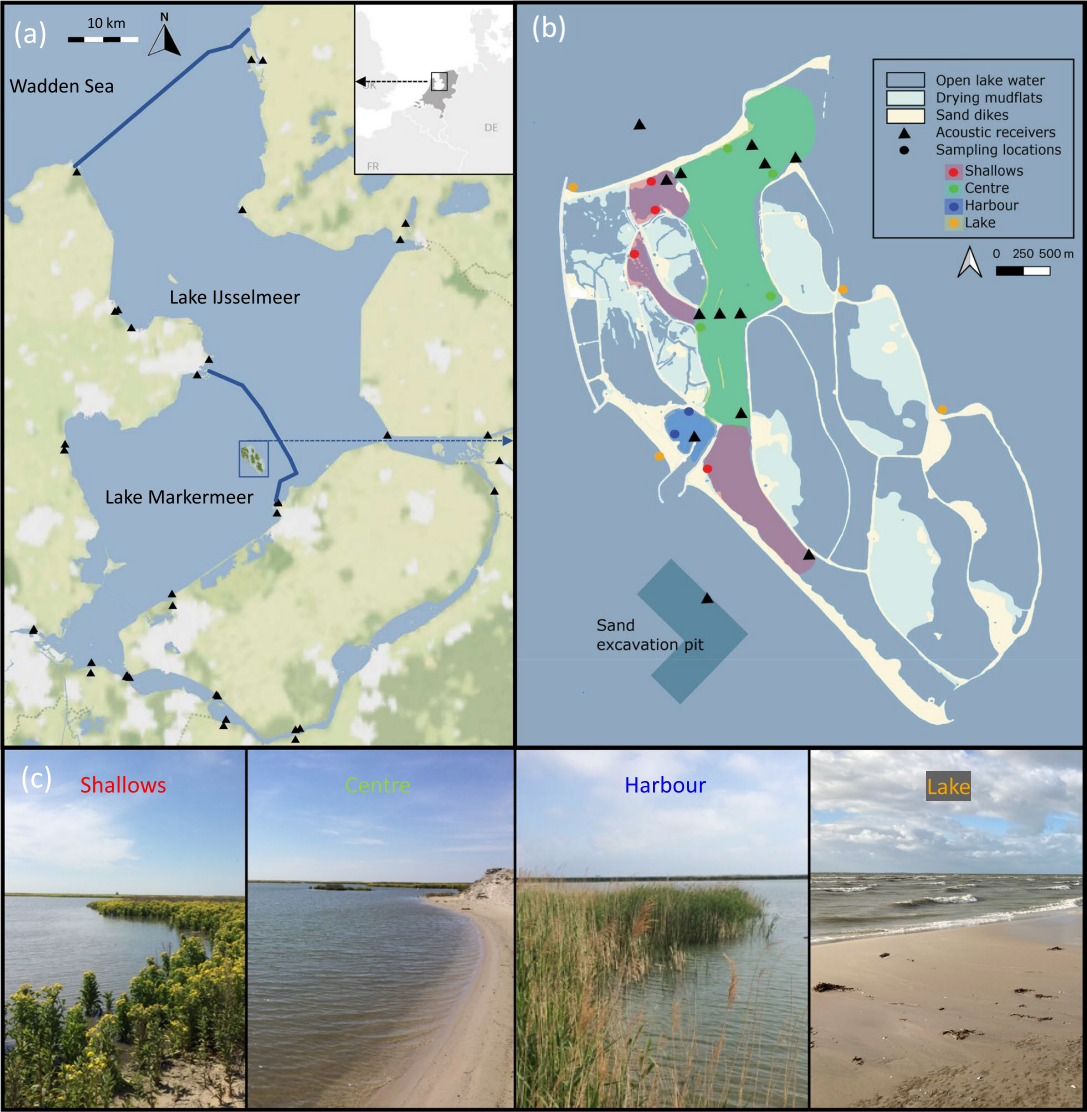
**a** The large-scale study area in the centre of the Netherlands (see inset) consisting of several connected waterbodies, including the focal lake Markermeer. Fish were monitored at large spatial scales through a network of acoustic receivers maintained by collaborators (n = 33, black triangles) throughout the lake system. The Marker Wadden archipelago is constructed in lake Markermeer, west of the ‘Houtribdijk’ dam that separates lake Markermeer and lake IJsselmeer. The Houtribdijk forms an incomplete boundary for fish with sluices at either end. **b** The Marker Wadden archipelago with sheltered gradual land–water transition shorelines leading into shallow littoral habitat, and the 30-m deep sand excavation pit created during the building of the archipelago on the southwest side. Fish were monitored at local spatial scales by a network of 13 acoustic receivers especially deployed for this study (black triangles). This created four sections varying in shelter and water depths; **c** Illustrative ground pictures of the four types of habitat indicated in panel **b** and as described in the methods

To address this knowledge gap, we capitalised on a large-scale restoration project in a strongly human-modified freshwater ecosystem, lake Markermeer in the Netherlands (Fig. 1a). Lake Markermeer is a Natura 2000 protected large shallow lake with a uniform water depth of 4 to 5 m [43, 49]. This temperate lake has been formed by the construction of two large dikes in a former marine estuary of the River Rhine over the last century, which created two freshwater lakes: lake IJsselmeer of 110.000 ha and lake Markermeer of 70.000 ha. While lake IJsselmeer still receives the original riverine influence from a branch of the River Rhine (River IJssel), lake Markermeer is almost a landlocked lake with water retention times up to 12 months, regulated water levels, and restricted possibilities of fish to move in and out of the lake via sluices [14]. The combination of a uniform 4-m water depth, easily resuspended fine sediments, prevailing strong winds and long fetch lengths causes high turbidity levels in lake Markermeer. This limits light availability for primary production by macrophytes and benthic algae in the majority of the lake [32, 36, 75]. The lake is enclosed by steep rip-rap shorelines for water safety and therefore entirely lacks a natural littoral zone with gradual land–water transition zones [71].

The food web of lake Markermeer is relatively simple due to the artificially created homogeneity in abiotic conditions. Partly due to this homogeneity, fish and bird densities and diversities have been declining over the last decades [68, 72], which induced a large-scale restoration project. In 2016, the project called “Marker Wadden” was initiated, which added land–water connections, shelter, shallower and deeper waters to the lake. The aim was to stimulate the food web bottom-up by reducing the abiotic and biotic homogeneity (for more background see [43]). Because no historic reference situation was available, the project adopted a forward-looking restoration approach by adding habitat that had never been present in the lake before, and the question arose how biota would respond.

The Marker Wadden project created an archipelago of 1000 ha in the lake from local clays and sands (Fig. 1b, c). Five islands were built as ring dikes from Pleistocene sands, and filled sequentially with layers of Holocene fine clays and silts that were allowed to subside between fillings. Waters between the islands were sheltered from the strong wind and wave action in the lake, and partly shallowed from the original lake depth of 4 m to less than 2 m deep. The archipelago started providing a heterogeneous new area with sheltered waters of varying depths surrounded by shorelines that varied in slope, sediment type and establishing vegetation. Because the sediment for construction was extracted locally, three deep sand excavations of up to 30 m deep were created within a kilometre radius of the archipelago. The newly created area was expected to become valuable habitat for the fish and bird communities in the lake [19, 22, 38].

Here, we aimed to quantify habitat use of five important fish species of two different feeding guilds in relation to the new creation of sheltered shallow littoral zones and deep sand excavations in the shallow lake with a uniform water depth. We quantified adult fish movements at local spatial scales and across the larger system of multiple lakes. We hypothesized that (1) new sheltered littoral zones would form attractive spawning and feeding habitats for both piscivorous and omnivorous fish species, (2) at the local scale, movements through the newly created shallower and deeper habitat types would show seasonal and feeding guild-specific variation, and (3) at the lake-wide scale, the archipelago would provide a relatively important year-round residence area for all five species because it offers sheltered habitats of varying water depths. We tested our hypotheses by monitoring movements of 78 adult fish of five important species in the system via acoustic telemetry over one year, combined with quantifying densities of aquatic macro-invertebrates as proxy for food availability for omnivorous species and quantifying densities of young-of-the-year fish as proxy for food availability for piscivorous species. We tracked the fish with high spatial resolution at the local spatial scale of the archipelago via a specially deployed acoustic telemetry receiver network designed for this study (receivers every several hundred metres), and obtained information on large scale fish movements through the large lake-system via receivers of a longer-term network maintained by our institutions (receivers tens of kilometres apart).

## Materials and methods

### Study area

We studied fish movements throughout a large system of several human-created lakes in the former marine estuary of the River IJssel in the Netherlands, called the Zuiderzee [13]. For water safety purposes, this estuary was separated from the Wadden Sea in 1932 by the Afsluitdijk and further divided into two lakes in 1975 by the Houtribdijk, which created two large lakes (Fig. 1a). During the twentieth century several land reclamations reduced the total surface area in the system by about 40% and compartmentalized the lake system via water level regulating partial barriers such as sluices [14, 71]. This study concentrates on the oligo-mesotrophic lake Markermeer, of which particularly the eastern wind-exposed side is characterized by strong resuspension of fine sediments that limit light availability for primary production. To maximize potential effects on the lake, the new 1,000-ha archipelago has been constructed in the wind-exposed northeast as outlined in the introduction (Fig. 1b).

Between the islands of the archipelago several lagoons of varying depths were constructed to create sheltered conditions with shallow waters where macrophytes could establish [31, 32, 43]. The archipelago included a sheltered harbour bay with the original depth of the lake, and a large 30 m deep sand excavation off the southwestern shore from which sediment for construction of the archipelago was extracted. Sand was additionally extracted from two other smaller and less deep sand excavations farther away from the islands. At the time of this study in 2020, turbidity at the archipelago was still high, potentially partly because the sediment had not settled yet after the building activities. Marsh vegetation had already established on the sheltered land–water transitions [43, 67] but macrophytes were only present at low densities [32]. The archipelago therefore provided fish with sheltered and shallower waters with vegetated shorelines, deeper sand excavations and a first phase of macrophyte establishment.

### Habitat types created at the archipelago

The construction of the archipelago created a mosaic of new habitats, of which we here distinguish habitat types that we refer to as “sections” (Fig. 1c). The sections were characterized mainly by the amount of shelter and the water depth as follows: (1) the “shallows” sections consisting of shallower waters with water depths < 1 m, in total ~ 50 ha, and fine clays sediments with high organic matter contents. Dominant vegetation types in these sheltered areas were *Tephroseris palustris* and *Epilobium hirsutum*; (2) the “centre” section of ~ 80 ha, consisting of sheltered waters with depths up to 2 m and surrounded by dikes constructed of Pleistocene sands with low organic matter contents. Little vegetation developed in these waters or on the shorelines; (3) the “harbour” section, a ~ 8 ha sheltered bay with the lake’s original water depth of 4 m, surrounded by a reed belt shorelines of 2 to 3 m wide *Phragmites australis*; and (4) the open water of the lake, which is characterized by exposed shorelines without vegetation. As a fifth habitat type, we considered the main 30 m deep sand excavation off the exposed western shore of the archipelago (Fig. 1b).

### Young-of-the-year fish and macro-invertebrate sampling

We assessed variation in young-of-the-year fish densities as a proxy for a recruitment function and food availability for piscivorous Percidae, and macro-invertebrate dry biomass as proxy for food availability for omnivorous Cyprinidae. From May to August 2020, we sampled young-of-the-year fish communities monthly at 14 locations outside and inside the archipelago. Young-of-the-year fish densities were assessed using a multi-gear sampling strategy that targeted multiple fish species and sizes [20]. We used this to calculate one catch-per-unit-effort (CPUE) for each location per month. We used two types of dipnets to catch fish along the shoreline in water up to 1 m deep and between the marsh vegetation, and one seine net to catch faster larger fish farther from the shoreline. Dipnet 1 was 21 × 21 cm, with a 0.5 mm mesh, used over a 50 m track, resulting in a sampling volume of 2.2 m^3^. Dipnet 2 was 55 × 70 cm, with a 3 mm mesh, used over 50 m, resulting in a sampling volume of 19 m^3^. The seine net was 15.0 m long, 2.5 m heigh, with a 5 mm mesh from knot to knot, which we used from a boat over a distance from the shoreline 20 m. Because the net reshaped upon use, its effective fishing width became 8 m and height became 1.5 m—leading to a sampling volume of ~ 240 m^3^.

Captured fish were counted, identified, total lengths measured to the nearest mm below in the field, and released. In case of excessive amounts of fish of the same species, total lengths were determined on subsamples of minimally 30 fish for logistical reasons. Larvae that were too small to identify in the field (< 20 mm) were stored on 4% formalin solution (Steve1) and identified within six months in the laboratory under a Leica 205C stereomicroscope based on the key of Pinder [53]. Only young-of-the-year fish were selected for analyses by making use of species-specific cut-offs for total length based on length-frequency distributions from previous Markermeer datasets (e.g. [78], which were 8937 of total 9782 captured fish, 91%). Sampling always occurred during daytime and at windspeeds with a maximum of 4 Beaufort.

Densities of pelagic macroinvertebrates in the water column were assessed in the same months and locations by placing a metal cylinder that was open at both ends (ø 50 cm, 50 cm high) on the sediment, within 5 m from the shoreline in water that was on average 24 cm (± 6SD) deep. The cylinder created an enclosed 0.2 m^2^ area in the water from which macro-invertebrates could no longer escape. Then we used a small dipnet (13 × 10 cm, 1 mm mesh size) to catch all macro-invertebrates present in this enclosed water. Densities of benthic macro-invertebrates in the sediment were assessed by sieving a sediment core (ø 6 cm, 10 cm deep) taken in the cylinder over a 0.72 mm mesh sieve. Both samples were stored separately on 70% ethanol at 4 °C until identification and total body lengths were measured to the nearest mm in the laboratory under a Leica 205C stereomicroscope within six months. A proxy for total biomass of macro-invertebrates was calculated by calculating macrofauna dry weights based on published length–weight relationships of these groups derived from Appendix S1 in Méthot et al. [47], and as assessed for *Neomysis* sp. by Aaser et al. [1]. Data from the metal cylinder and sediment core was combined to one estimate of macro-invertebrate dry biomass per 0.2 m^2^.

### Acoustic telemetry

We selected five dominant fish species in lake Markermeer for acoustic telemetry [78]: three Cyprinidae species (common roach *Rutilus rutilus*, common bream *Abramis brama* and ide *Leucistus idus*) and two Percidae species (pikeperch *Sander lucioperca* and European perch *Perca fluviatilis*). The Cyprinidae species are all considered omnivorous during their adult life stages in this study system, as they are feeding predominantly on benthic and pelagic macroinvertebrates. However, depending on food availability, these omnivores can also feed on zooplankton, filamentous algae and macrophytes [6, 23, 29]. The Percidae species are predominantly piscivorous when they are mature [24, 28, 37]. All five species spawn in spring and can extensively migrate between shallower littoral and deeper pelagic habitats, and among foraging, overwintering and spawning areas [8, 15, 24, 30, 39, 79]. Important drivers for movements include access to food, safety from predators, water depth, and water temperature [10, 61, 62, 79].

All adult fish were captured using a seine net in the harbour of the archipelago (Fig. 1b) during spring 2020. The seine net was 375 m long, 6.5 m high, and mesh sizes were a combination of 40, 18 and 15 mm (finer mesh sizes at the cod-end). Fishing occurred during three sessions: on February 10th, March 8th and April 9th, with three hauls per day. Captured fish of the five target species were identified and measured for total length to the nearest mm (measuring only subsets in case of excess fish).

Ninety fish captured during March and April (20 pikeperch, perch and roach; 18 bream and 12 ide) were selected for the tagging study. These fish were measured for their total length to the nearest mm and their body mass to the nearest gram on a balance (d = 0.1 g). The sizes of the selected Cyprinidae ranged from 21.5 cm to 61.7 cm (mean: 31.5 ± 8.5 SD). The sizes of the Percidae ranged between 23.3 cm and 53.5 cm (mean: 34.6 ± 10.0 SD). The fish were sexed and checked for gonad stages based on swollen abdomen, or milt or roe appearing from the cloaca after a gentle squeeze of the abdomen, and in the case of male Cyprinidae, the presence of mating tubercles. Fish were anaesthetized by placing them in a separate tank with a 2phenoxyethanol 0.4 ml L^−1^ solution until no muscle tension could be felt. Fish were then placed upside down in surgery cradles and a continuous 2phenoxyethanol solution was applied to their mouths. A mid-ventral incision was made, and fish were fitted with acoustic transmitters model V9-2L manufactured by Vemco Ltd, Halifax, Canada. Cavities were closed using two sutures (soluble Vicryl from Ethicon, New Jersey, USA). Fish were left to recover in a recovery tank and released when active swimming behaviour was restored. The V9 tags were programmed with a nominal delay of 60–120 s (mean every 90 s), with an estimated battery-life of 346 days.

To monitor the fish with high resolution at the local spatial scale of the archipelago, we deployed a network of 13 acoustic receivers of type VR2W (Vemco Ltd, hereafter “receiver”) throughout the study area (Fig. 1b). The receivers formed arrays that divided all waters within the archipelago into the previously described and pre-defined sections (Fig. 1b), which we used to calculate residence times in the different sections. Additionally, movements at the larger spatial scale of the lake system were monitored through an already existing network of 33 receivers that were placed strategically at locations where the fish had to pass in order to move from one compartmentalized part of this system to another (Fig. 1a), or at locations where they could enter into a river or polder. Hence, whereas the detection ranges of the receivers (maximally 500 m based on a range testing, and depending on e.g. water depth and weather conditions) was limited in comparison to the scale of the lake system, this network was suitable for a general assessment of large-scale movements over distances over tens of kilometres of fish moving amongst water bodies, and to verify that fish no longer observed in the local network at the archipelago had indeed left the archipelago.

Two of the receivers at the archipelago were deployed later than the start of the study on the 20th of May 2020, and were placed 500 m outside of the archipelago, (Fig. 1b). The southwestern receiver was placed at the main 30-m deep sand excavation that was created close to the archipelago for its construction. The northern receiver was placed on a featureless 4 m-deep section of open water as a representative of the open water (Fig. 1b).

### Statistical analyses

Effects of habitat section on young-of-the-year fish densities were tested using in month-specific generalized linear mixing models with CPUE as dependent variable depending on habitat section as factor, modelled with a Poisson error distribution with log link function (package lme4, [4]). Because we were interested in variation among locations, we removed the zero-catches across all locations for the two dipnets in July and August. Differences in macro-invertebrate biomass were tested in a similar model, but modelled with a zero-inflated Tweedie distribution to account for zero-catches (package glmmTMB, [9]). In all models, sampling location within each habitat section was included as random intercept to account for variation among locations. To determine statistical differences among the habitat sections, Tukey’s honest significance tests were computed using package “emmeans” [44]. Quantile–quantile plots and Shapiro-Wilks tests were used to check normality of model residuals and factor significance was determined through comparisons of full models with null-models.

To test whether fish species differed in their spatio-temporal habitat use we performed a residency analysis with package Actel in R [21]. In this analysis, we distinguished the different sections at the archipelago (i.e. shallows, centre, harbour, lake and the sand excavation) and calculated for each fish how many hours it was resident in each section based on passage times of the fish through the deployed arrays (Fig. 1b). To exclude false or erroneous detections, a minimum number of two detections was needed before a fish was assigned to a section. Fish with a continuous presence at one receiver > 30 days were conservatively removed because this indicated possible death or could not be trusted to accurately represent fish behaviour. After filtering, data for 78 individuals were further analysed. To assess the relative attractiveness of the main deep sand excavation as a local habitat feature, we additionally compared the number of detections and the number of individuals on the two receivers just offshore of Marker Wadden using species-specific chi-squared tests of independence.

To test whether the archipelago would be relatively important residence area for adult fish compared to other locations in the lake at larger spatial scales (hypothesis 3), we assessed how long tagged fish remained in the study area after release and how they moved through the lake system at larger spatial scales. We first tested fish residence times at the archipelago using a Kaplan-Meijer survival analysis in which time spent after release was the dependent variable and family or species as predictor variables. A fish was considered to have been resident in the area between its first and last detection on any of the 13 receivers within the study area (i.e. including the two receivers within 500 m of the archipelago). For a more detailed assessment, additionally a Cox proportional hazards model was fitted to assess effects of species and spawning readiness upon catching as predictor variables using a log-rank survival method. Gender was disregarded as an independent variable because it could not reliably be assessed. A Fisher’s exact test was used to compare the likelihood of detection outside of the study area between fish families. Results were considered significant at *P* ≤ 0.05. All statistical analyses were performed using R for Statistics [55].

## Results

### Spawning and feeding habitat

Adult fish densities of the five focal species increased at the archipelago during early spring. During three hauls of seine netting for adult fish on the 10th of February 2020 we only captured six individual fish > 20 cm of the five focal species. During repeated sampling with equal effort on the 8th of March we caught slightly more adult fish of the focal species: four perch, 16 pikeperch, seven ide, seven roach and two bream > 20 cm. On the 10th of April fish numbers of the focal species had increased to catching more than 60 individuals per species with the same effort (Additional file 1: Table S1). In March and April, gonad stages were checked for 90 fish of the focal species selected for the tagging study, which indicated that 33 (37%) were ready to spawn (Table 1).

**Table 1:** Background information on the 90 individual fish of the five target species that were used in the telemetry study

Species	*Abramis brama*	*Leuciscus idus*	*Rutilus rutilus*	*Perca fluviatilis*	*Sander lucioperca*
Initially tagged fish	18	12	20	20	20
Successfully tracked fish	16	10	18	15	19
Mean total length in cm (SD)	39.8 (9.3)	28.1 (2.0)	26.1 (2.3)	26.1 (2.1)	43.5 (7.0)
Gonad stage for spawning	1	0	12	11	9
Fish detected outside archipelago	11	6	6	1	7
Fish returning after once leaving archipelago	4	5	2	0	5

**Fig. 2:**
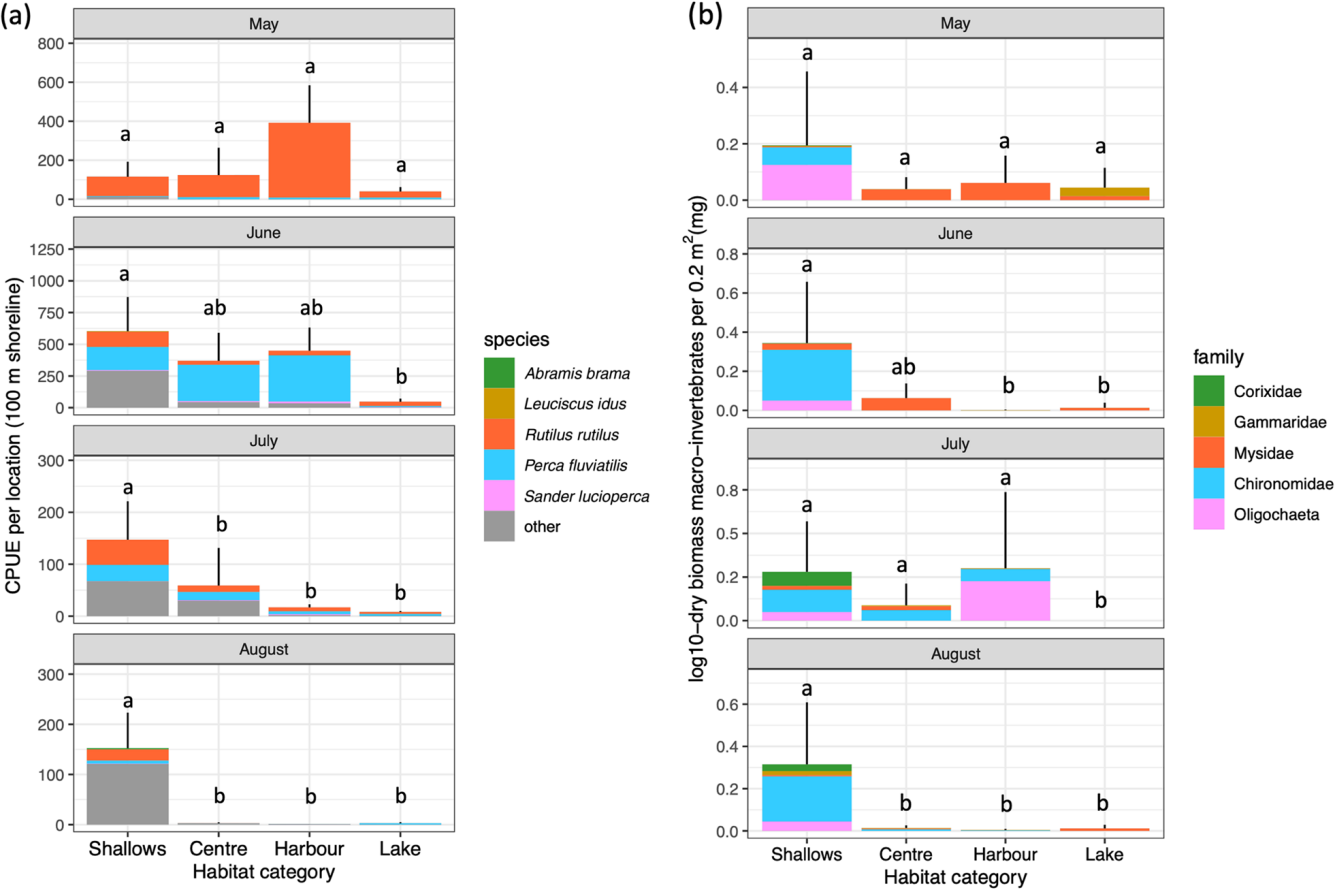
**a** Young-of-the-year fish CPUE per 100 m shoreline sampled within the four habitat categories within the archipelago waters and along the exposed open lake shores. All sites were sampled from May until August, error bars denote standard errors across the sampled locations. Proportion of CPUE per focal fish species is indicated by colours. Letters denote significance of differences among habitat types based on generalized linear models (statistical details in Additional file 1: Table S2a). **b** Biomass of benthic and pelagic macro-invertebrates per surface area (0.20 m^2^), for the four categories of shorelines on a log10-scale. The proportional contributions of five macro-invertebrate families to the biomass are overlaid on the bars, which together represent 98.9% of all organisms caught. Letters denote significance of differences in total biomass among habitat types based on generalized linear models (statistical details in Additional file 1: Table S2b)

Young-of-the-year fish of 12 different species were captured during all sampling efforts in 2020, which included young-of-the-year fish of the five focal species (Fig. 2a). Additionally, we captured young-of-the-year fish of four native species (*Alburnus alburnus, Gasterosteus aculeatus, Gymnocephalus cernua* and *Osmerus eperlanus*) and three non-native species (*Neogobius fluviatilis, Ponticola kessleri, Neogobius melanostomus*) in lower numbers. Densities were highest for perch and roach, and most of the season higher in the three habitat types inside the archipelago than along the unsheltered shorelines of the open lake (Fig. 2a). The shorelines of the harbour section with helophyte vegetation contained particularly high densities of fish larvae in stage 1 and stage 2 (sensu [53]).

Pelagic and benthic macro-invertebrate biomass was also higher in the three sheltered inside shorelines than in the unsheltered outside shorelines during most of the season (Fig. 2b), although species composition varied among habitats. Macro-invertebrates were dominated by Chironomidae larvae and Oligochaeta in the sheltered sediments, and *Neomysis* sp. (Mysidae), *Gammarus* sp. (Gammaridae) and Corixidae in the water column close to the sediment. In total 468 individuals were caught, of which 463 (98.9%) belonged to one of these five families. Other identified taxa were Corophidae and Leptoceridae.

### Local-scale fish movements

**Fig. 3:**
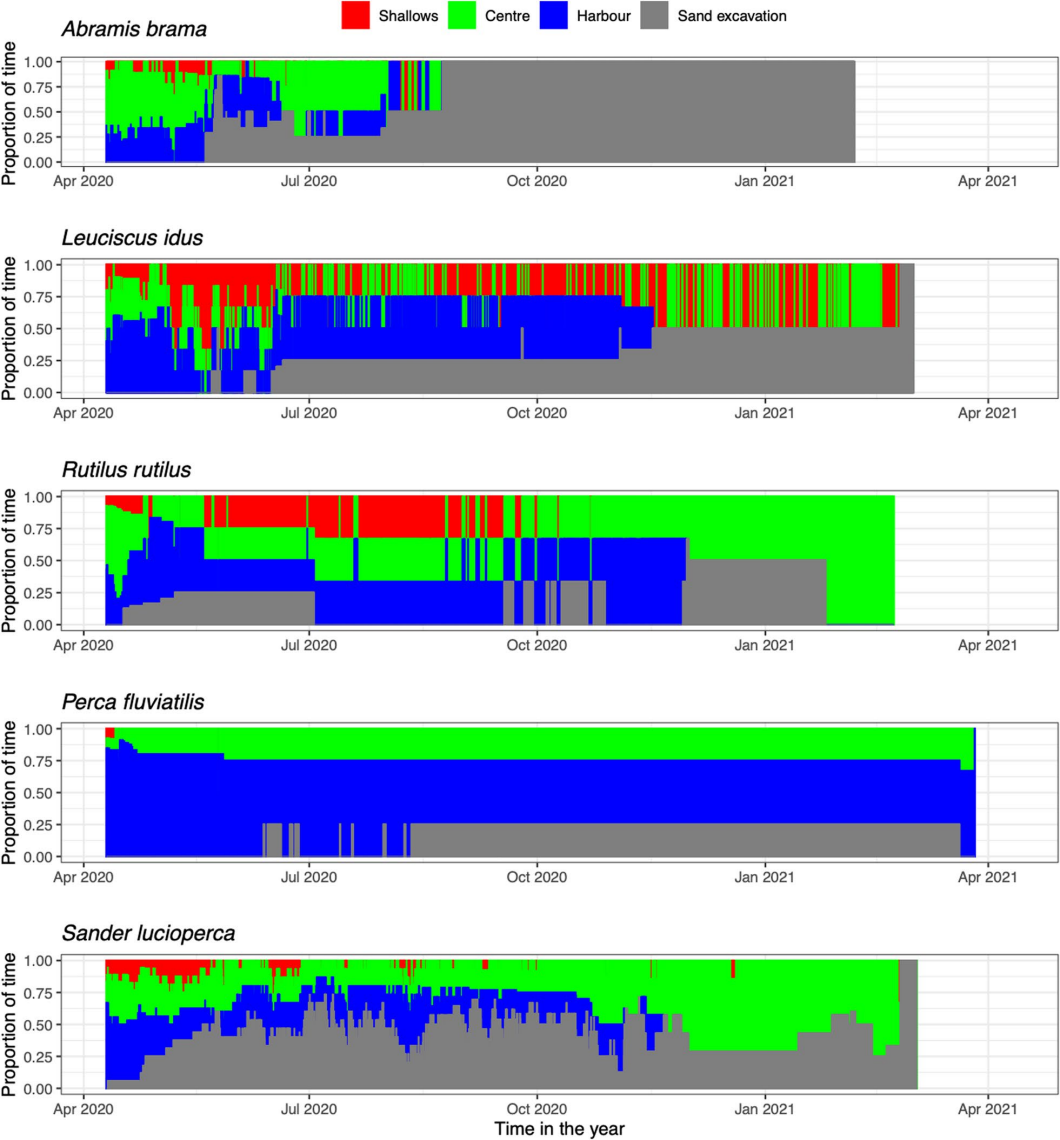
Adult fish habitat use for the five study species at local spatial scales of the Marker Wadden archipelago, depicted as proportion of time spend at each location type (see Fig. 1b) over the year that the fishes were monitored. Note that the sections differed in total surface areas with the harbour being the smallest areas (~ 8 ha) followed by the marshes (~ 50 ha) and the centre as largest area (~ 80 ha); and note that the numbers of individuals on which the data are based declined over time in the season (see Fig. 4)

The shallow vegetated marsh zones of the archipelago were mostly visited early in the summer season (Fig. 3), with only sporadic use later during the summer and winter. Throughout the year the 2 m-deep centre section was visited by all species. The location where the fish were captured and released, i.e. the archipelago harbour, remained a relatively attractive habitat throughout the year despite its small size compared to the other sections. Later during the summer most of the fish had left the archipelago completely (see next section), and the fish that remained were still most often found in the harbour section or the 30 m-deep sand excavation off the southwestern shore.

The main sand excavation was visited continuously during the entire monitoring period that the receiver was deployed (May 2020–Feb 2021), mainly by pikeperch (Fig. 3). Significantly more individual fish were detected over the entire duration of the study near the sand excavation (37%, 29 of 78) than by the receiver at the open lake (15%, 12 of 78, *Χ*^*2*^ = 8.47, df = 1, *P* = 0.004). The number of detections by the receiver close to the sand excavation (17,272 counts) was > 50 × higher than the number of detections by the northern receiver (333). The difference in the number of detections was significant for bream (*Χ*^*2*^ = 467.4, *P* < 0.001)*,* ide (*Χ*^*2*^ = 151.04, *P* < 0.001)*,* perch (*Χ*^*2*^ = 2051, *P* < 0.001) and pikeperch (*Χ*^*2*^ = 15,536, *P* < 0.001). No difference in the number of detections was found for common roach. Pikeperch accounted for 89.95% of all detections on the sand excavation receiver and 63% (12 of the 19) of the individuals were detected at this receiver.

### Large-scale fish movements

**Fig. 4:**
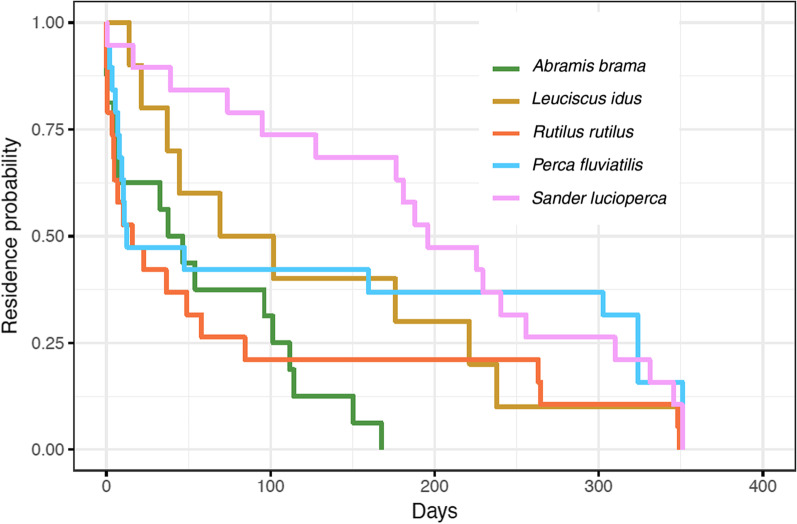
Kaplan-Meijer survival curve displaying the number of days between the first and last detections of three Cyprinidae species and two Percidae species in the area (n = 78). The vertical axis displays the portion of the fish species still considered resident in the shallow sections of the archipelago (i.e. excluding visits to the sand excavation only). Percidae remained significantly longer around the archipelago than the Cyprinidae (Survival Analysis at the family level: *P* = 0.015, statistical details per species level in Additional file 1: Fig. S1)

**Fig. 5:**
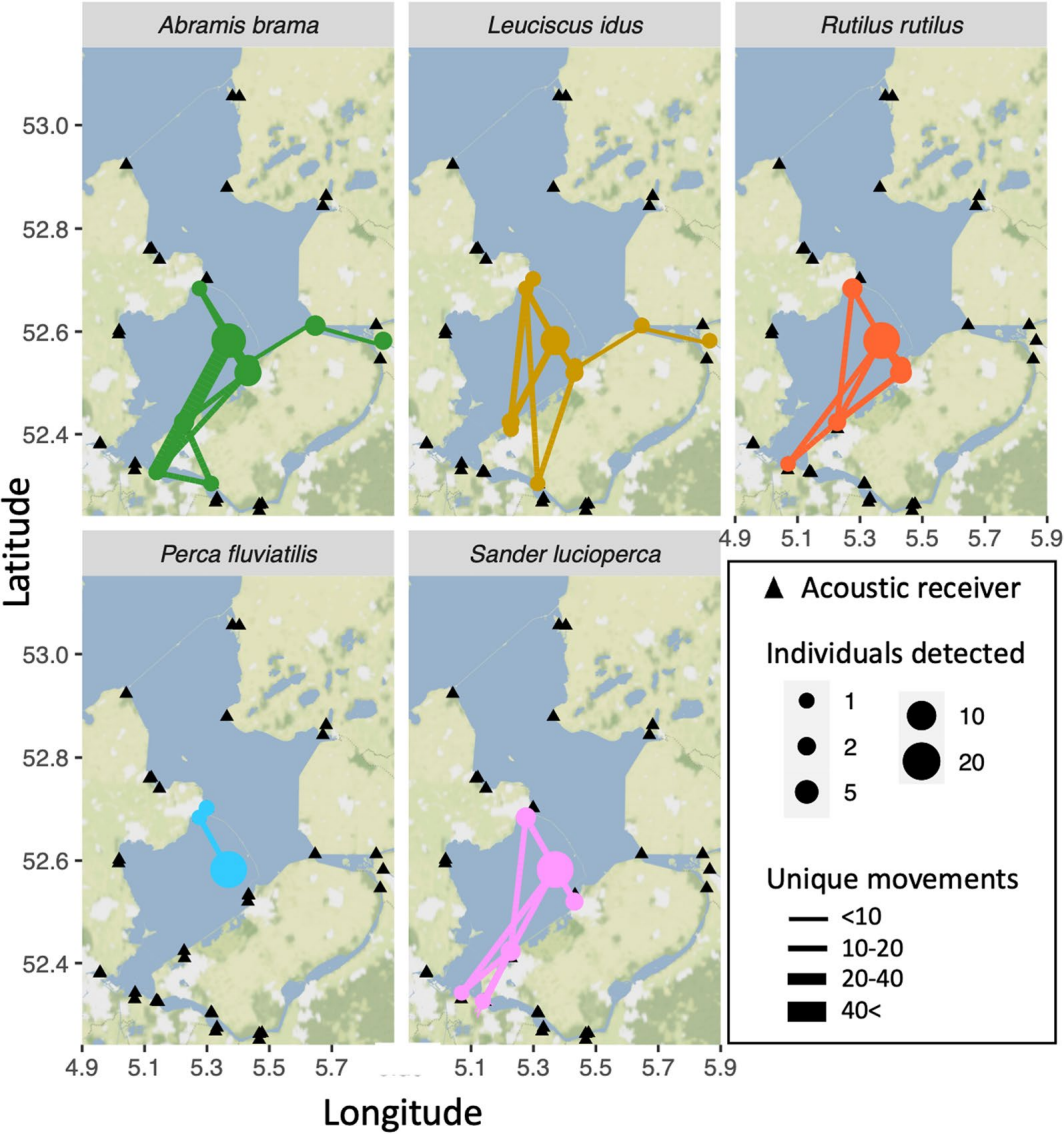
Lake-scale movements of adult fish of the five studied species in the large-scale study area with the network of receivers indicated with black triangles. Coloured circles indicate receivers that were visited by fish. The size of the circles is scaled to the number of detected individual fish. The widths of the connecting lines depict the number of unique movements between receivers for all fish of a species combined

Percidae remained resident at the Marker Wadden archipelago for a longer period than Cyprinidae (median Cyprinidae 37.4 days, median Percidae 143.6 days, Kaplan-Meijer survival analysis, Fig. 4, Additional file 1: Fig. S1a, *P* = 0.015). The log-rank based Cox proportional hazard model revealed a similar pattern at the species level, with some species-specific variation (Additional file 1: Fig. S1b. Fish displaying visual signs of spawn readiness left the archipelago significantly sooner than fish not displaying such signs (*P* = 0.005, Additional file 1: Fig. S1b). In line with a reduction in fish detections at the local receiver network, 31 (40%) of the 78 successfully tracked fish were detected by the extended network of receivers outside of the archipelago (Fig. 5). Of these 31 fish, 16 (52%) returned to the study area at least once (Table 1). Cyprinidae were significantly more likely to be detected outside of the study area than Percidae (*P* = 0.027).

## Discussion

Newly created sheltered littoral habitats and a deep sand excavation formed attractive habitat features for adult freshwater fish in an ecologically degrading shallow lake. Habitat use varied among the types of created habitat, among fish species, and over the season. Adult fish of the five focal species of this study moved towards the newly created shallow sheltered habitats in early spring, which included both omnivorous and piscivorous species, of which many individuals were ready to spawn. During the subsequent productive season, high densities of young-of-the-year fish appeared in the shallow waters along the inner shorelines of the archipelago. Young-of-the-year fish of common roach and European perch were the most abundant, suggesting that their spawning was most successful or the survival of their larvae was the highest.

A broad-scale fish survey in the archipelago during different seasons of 2020 [69] showed that—besides our five focal species—13 other fish species also used the Marker Wadden archipelago. In total, 18 of the 23 species known to occur in Lake Markermeer were detected during these surveys. The new sheltered shallow waters formed productive spawning and recruitment habitats in the lake, in which we captured the highest densities of young-of-the-year fish in the most sheltered, shallow vegetated waters. Although we have no knowledge about the young fish densities that might have been present in the open water of the lake prior to the creation of the archipelago, the densities of young-of-the-year fish and macro-invertebrates were much higher in the sheltered habitats than along the newly created wind- and wave exposed shorelines. This shows that macro-invertebrate and young-of-the-year fish densities were stimulated more by the creation of shallow sheltered habitats than by the creation of new littoral zones still exposed to wind- and wave actions. This knowledge extends previous studies on how creating new littoral zones affects other trophic levels of the aquatic food web [31–33, 43].

### The functionality of the new habitats at the local scale

At local spatial scales, the archipelago offered a mosaic of different habitats. Shorelines ranged from exposed sand banks leading into deeper water to sheltered gradual land–water transitions with emergent vegetation. The key contrasts with the open water of the lake were the created shelter from wind- and wave action, shallower waters, deeper sand excavations, and the establishment of low densities of submerged and emergent vegetation such as *Potamogeton* spp., *Myriophyllum spicatum*, *Phragmites australis*, *Typha latifolia* and *Tephroseris palustris* [32, 43, 59]. These sheltered waters likely attracted adult fish of our five focal species at the onset of the spawning season, because initially low densities of adult fish during winter increased manyfold in early spring. Adult fish that are ready to spawn typically search for sheltered waters, stable sediments, shelter from wind-exposure and littoral vegetation for spawning [18, 35]. The newly created littoral zones are typical examples of suitable habitat for young-of-the-year fish [50]. Although not all the arriving adult fish showed clear signs of spawning (yet)—and they may have had other reasons for visiting the archipelago—reproductive activity of multiple adult fish species was evidenced by the high densities of young-of-the-year fish as the season progressed. This included yolk-sac larvae of only a few days old.

We expected the different sections of the new area to vary in their importance for young-of-the-year fish, as determined by the abiotic conditions [18]. We found that the densities of young-of-the-year fish and macro-invertebrates were highest along the sheltered shorelines with the shallowest water and shortest wind fetch lengths. Larval densities were particularly high for roach in the harbour section in May, which may be related to the combination of sheltered conditions, an extensive reed belt as potential spawning substrate, and relatively safe deeper water in the vicinity for the spawning adult roach. Later during the season, young-of-the-year fish of perch and roach dominated the species composition, and the small fish concentrated in the shallowest vegetated shorelines. The strong concentration of yolk-sack larvae in the reed belts surrounding the deeper harbour section suggests that most spawning activity took place in this harbour, and that larvae thereafter autonomously moved into the shallow habitats nearby to feed on the higher concentrations of zooplankton [33]. However, this idea requires further research.

Direct spawning in the shallow sections was also possible (as observed for carp *Cyprinus carpio*, pers. obs. CvL), but densities of adult fish were not specifically high in these areas in spring. Instead, adult individuals of both feeding guilds tended to avoid the shallow waters (< 2 m) during their presence at the archipelago. Although some individuals of roach and ide occasionally moved into the shallow areas (Fig. 3), both feeding guilds spent most of their time in the deeper sheltered harbour of the archipelago. This harbour was also where the fish were initially captured, tagged and released, so this could be an effect of residency, and future studies with fish captured at multiple locations in the study system would be needed to overcome this bias. However, fish of all species still frequently moved from the harbour into the 2 m-deep centre section of the archipelago and the deep sand excavation nearby, after which they actively and frequently returned to the harbour. This suggests that the 4-m deep sheltered water body surrounded by reed belts offered a particularly interesting habitat type for spawning as well as residency.

Potential reasons for the fish to stay relatively often in the harbour is its 4 m water depth. Firstly, the 4 m water offers more protection against avian predators such as great cormorants (*Phalacrocorax carbo*) and marsh harriers (*Circus aeruginosus*) than the very shallow new waters. Although most piscivorous birds at the archipelago were species that depredate smaller fish (e.g. Eurasian spoonbill *Platalea leucorodia*, common tern *Sterna hirundo*) [16], solely the risk of predation can already determine the spatial distributions of freshwater fish [2, 61]. Particularly Cyprinid prey fish are well-known to adjust their habitat selection to the presence of predatory birds and larger piscivorous fish [7, 27, 58]. Secondly, the sheltered 4 m water is a relatively stable environment with little influence of potential water level fluctuations due to changes in wind direction. Like in most large lakes, the long fetch lengths in lake Markermeer can cause water to accumulate downwind and thus decrease or increase water levels within a few hours [51]. This implies that fish residing in very shallow waters—whether juveniles or adults—have to trade-off rewards of going into risky shallow waters (e.g. high food densities) with the potential costs (e.g. unable to escape shallow waters, or being depredated by piscivorous birds because of lack of deep water refuges). In the case of the new shallow waters at Marker Wadden, our study suggests that the rewards for adult fish to go into these relatively dangerous areas are lower than the potential costs, while this seems the reverse for young-of-the-year fish.

Percidae and Cyprinidae differed in their use of the new habitats at the archipelago. For piscivores Percidae such as perch and pikeperch, an important reason may have been to hunt on the high densities of juvenile fish [26]. Particularly the larger pikeperch is relatively safe from avian predators, and their vision makes them effective hunters during darker conditions and in the turbid waters that were created [12, 30, 34, 84]. Perch may similarly have used the littoral zones for hunting, but also used the area extensively for spawning. The shallow vegetated habitats were likely of lesser value as spawning habitat for pikeperch than for perch, as densities of young-of-the-year pikeperch were much lower and pikeperch typically builds nests in deeper waters [30, 42].

In contrast to the clear foraging opportunities for the two Percidae species, the food availability for the more omnivorous Cyprinidae species was not as clear. Four years after construction, the macro-invertebrate biomass was dominated by only five different taxonomic groups. Macro-invertebrate densities were much higher in the more shallow, warmer and productive areas than in the more exposed shorelines, which was in line with the observations of higher zooplankton and vegetation developments there [33, 43]. However, the macro-invertebrate densities elsewhere in lake Markermeer [73, 77] could still be up to ten-fold higher than the best sheltered shorelines of Marker Wadden in 2020. At least 17 different taxonomic groups of benthic macro-invertebrates are found throughout the lake at its larger scale [77], but in these first four years only the five macro-invertebrate groups colonized the new area with high densities. Due to the considerable spatial heterogeneity in benthic food availability in lake Markermeer, this may explain why the archipelago formed a relatively less attractive area for the omnivorous Cyprinidae than for the piscivorous Percidae. Many locations in the lake system will have offered equal or higher food availability for omnivorous fish than Marker Wadden. Although zooplankton densities were clearly higher in early summer for young-of-the-year fish, later during the season when Cyprinidae left the archipelago densities inside the archipelago and in the open water were very similar [33].

The observed species-specific variation in residency could have many other potential reasons than solely food availability, however, we expect at least a partial role for the here quantified difference in food availability. The lower attractiveness of the archipelago after the spawning season to Cyprinidae matched the observation that after the spawning season most of the adult Cyprinidae left the littoral habitats, while Percidae stayed (Fig. 4). Cyprinidae that left the archipelago made more extensive movements through the study system at larger spatial scales than Percidae (Fig. 5). Species like ide and bream are known for their high mobility in lake and river systems [8, 60, 82]. Together this suggests that the new shallow habitats were particularly important new habitat for Percidae, notably pikeperch.

### Functioning of the archipelago at the lake scale

The high variation in habitat types that the archipelago offers to fish made us hypothesize that it would be a relatively attractive location on the larger spatial scale of the whole lake system. Fish could benefit from local heterogeneity in water depths instead of moving to find these different habitat types only on larger spatial scales, after typically passing barriers such as sluices that consumes time and energy [70]. However, adult fish that left the archipelago after the spawning season only occasionally returned and seemed mainly attracted to the area for spawning. Based on our current data, return rates and habitat use throughout the non-spawning season was not higher than we could expect by chance (although notably pikeperch made extensive movements back and forth from the archipelago across lake Markermeer). Unfortunately, our tagging for 346 days did not fully include possible return for spawning in a subsequent spawning season, which would be interesting to further explore in future studies. We can therefore conclude that the archipelago served mostly as a transient reproduction location for Cyprinidae rather than a foraging or sheltering habitat. The conditions at the archipelago seem more suitable for Percidae and young-of-the-year fish than for adult omnivorous fish [19, 22, 38].

The construction of the archipelago also created three deep sand excavations, and we could quantify that the largest excavation just off the western shore of the archipelago was used by one-third of all tagged fish (Fig. 3). Compared to the receiver north of the archipelago that functioned as a “control” receiver in open water, the deep area attracted large numbers of fish that did not merely pass by but also remained stationary at the excavation. Especially pikeperch made extensive use of this deeper habitat, in line with their general preference for deeper areas [26, 40, 42], but also bream, ide and perch were detected more often at the sand excavation than by the receiver in the open lake. The explanation for the relative attractiveness of the deep sand excavation is currently not clear, however, it could be related to high densities of juvenile prey fish such as smelt *Osmerus eperlanus* seeking colder waters during summer, energy saving in calmer and colder water, increased safety against predation, or most likely a combination of these factors. Irrespective of the precise reason for the attractiveness of the deep areas, the sand excavation formed an attractive added feature in the homogeneous underwater landscape for fish in the lake.

### Implications

This study demonstrated how five common freshwater fish species responded within four years to large-scale changes in abiotic conditions. Addition of heterogeneity to uniform ecosystems like the human-created lake Markermeer can lead to a locally attractive area for fish. However, the use of the new habitat types varied among the five studied species and over their annual and life cycles, which can be used to predict the value and best-practises of new habitat restoration projects. As large-scale engineering projects are increasingly called upon to halt and reverse habitat degradation [43, 46, 52], our results provide an illustrative example of how large-scale abiotic changes in a habitat can have consequences for multiple species, feeding guilds and/or life stages of the same species. Specifically for the fish studied here, it predicts the habitat alterations may induce shifts in the fish community compositions on longer timescales, although the potential of the Marker Wadden project to have long-lasting effects on the fish community at the scale of the lake system presently remains unclear. We have shown how the long-term value of the new habitats will depend on the ecology of the targeted fish species, which predicts that any possible changes in the fish community will depend on the type of habitat that is created. Predicting the response of fishes to future habitat restoration projects in degrading freshwater ecosystems is urgently needed considering the degradation of fish communities and ecological integrity of freshwater ecosystems worldwide [64, 66].

## Supplementary Information

**Additional file 1:** Supplementary information file with background tables and figures. **Table S1**. Counts of individual fish of the five target species that were captured in the harbour section of the Marker Wadden with a 375 m seine net in different months of 2020. Only adult fish larger than 20cm are indicated. **Table S2a.** Statistical details on the models on CPUE per 100 m shoreline of the YOY. **Table S2b.** Statistical details on the models on biomass of aquatic macro-invertebrates. **Figure S1.** **a** Kaplan-Meijer survival curve displaying the number of days between the first and last detections of the five fish species per family in the study area (n = 78). The y-axis displays the portion of individuals still considered resident in the area. **b** A forest plot of a cox-proportional hazard analysis, performed at the species level and using the spawning likelihood of fish as a co-hazard. Significant deviation from the reference denotes an increased chance to leave the study area early

## Data Availability

The datasets generated during the current study are not yet publicly available due to ongoing further planned analyses, but will become available later and/or are available from the corresponding author on reasonable request.
