# Supplementary material for: Multispecies fish tracking across newly created shallow and deep habitats in a forward-restored lake
Source: Mov Ecol. 2023 Jul 27;11:43. doi: 10.1186/s40462-023-00405-1 (PMC10373381; doi:10.1186/s40462-023-00405-1)
Supplement: Supplementary file 1 — Additional file 1: Supplementary information file with background tables and figures. Table S1. Counts of individual fish of the five target species that were captured in the harbour section of the Marker Wadden with a 375 m seine net in different months of 2020. Only adult fish larger than 20cm are indicated. Table S2a. Statistical details on the models on CPUE per 100 m shoreline of the YOY. Table S2b. Statistical details on the models on biomass of aquatic macro-invertebrates. Figure S1. a Kaplan-Meijer survival curve displaying the number of days between the first and last detections of the five fish species per family in the study area (n = 78). The y-axis displays the portion of individuals still considered resident in the area. b A forest plot of a cox-proportional hazard analysis, performed at the species level and using the spawning likelihood of fish as a co-hazard. Significant deviation from the reference denotes an increased chance to leave the study area early [file 40462_2023_405_MOESM1_ESM.docx]

**Multispecies fish tracking across newly created shallow and deep habitats in a forward-restored lake**

Casper H.A. van Leeuwen, Joep J. de Leeuw, Olvin A. van Keeken, Joey J.J. Volwater, Ferdi Seljee, Roland van Aalderen, Willie A.M. van Emmerik, Elisabeth S. Bakker

**Supporting Information**

**Table S1**: Counts of individual fish of the five target species that were captured in the harbour section of the Marker Wadden with a 375 m seine net in different months of 2020. Only adult fish larger than 20cm are indicated.

|  | 10 February 2020 | 8 March 2020 | 9 April 2020 |
| --- | --- | --- | --- |
| *Abramis brama* | 2 | 2 | >120 |
| *Leuciscus idus* | 2 | 7 | >60 |
| *Rutilus rutilus* | 1 | 7 | >1500 |
| *Perca fluviatilis* | 0 | 4 | >60 |
| *Sander lucioperca* | 1 | 16 | >60 |

**Table S2a**: Statistical details on the models on CPUE per 100 m shoreline of the YOY


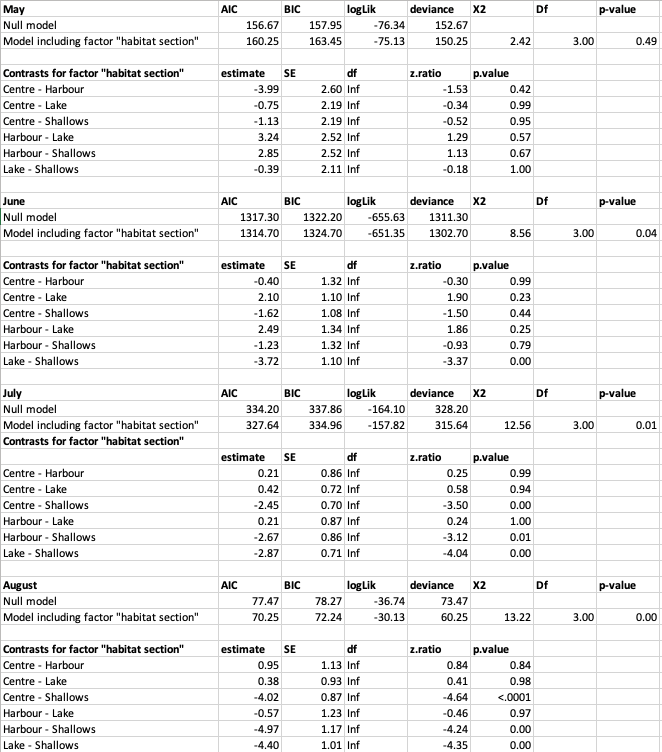
**Table S2b**: Statistical details on the models on biomass of aquatic macro-invertebrates


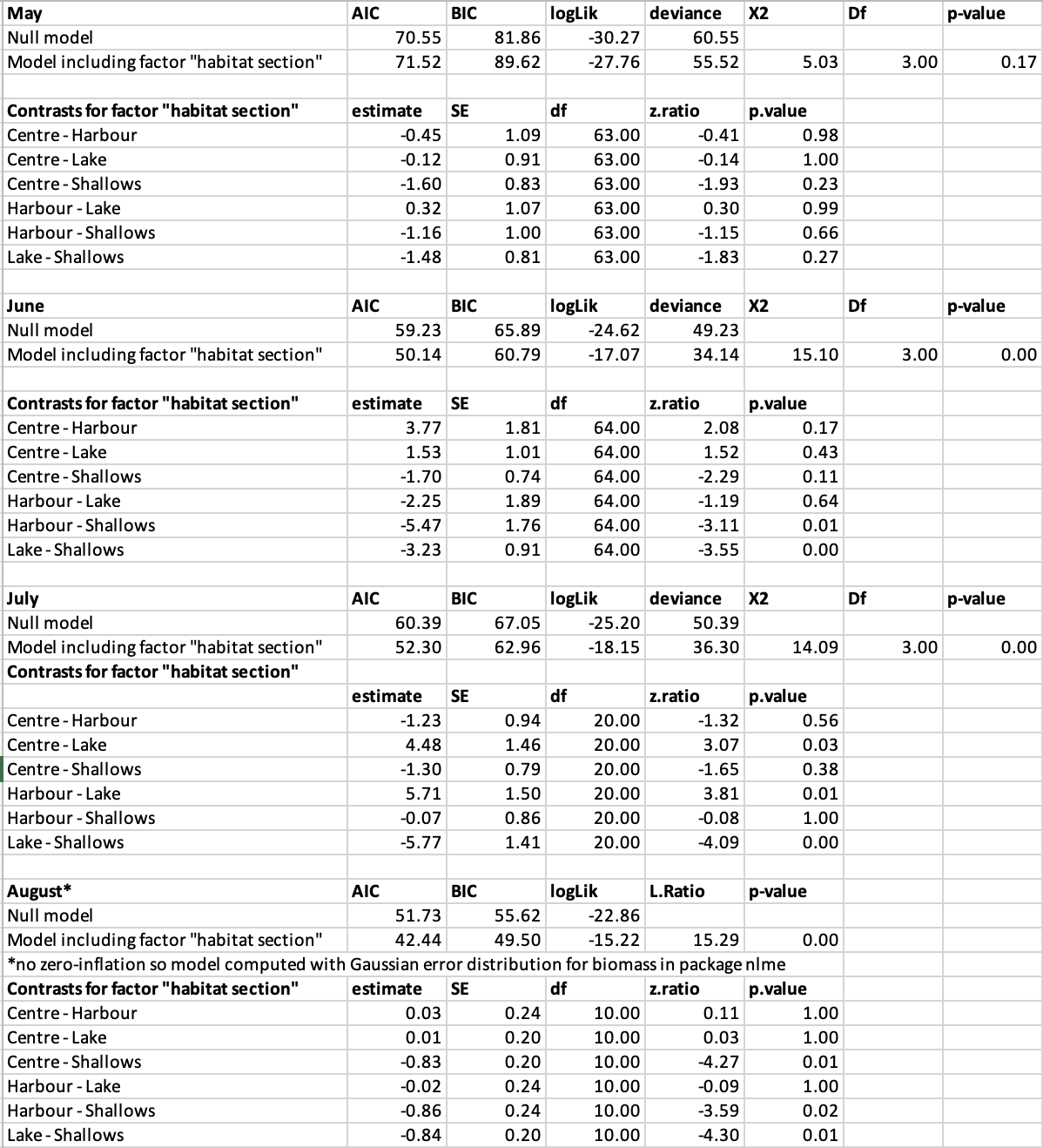


**
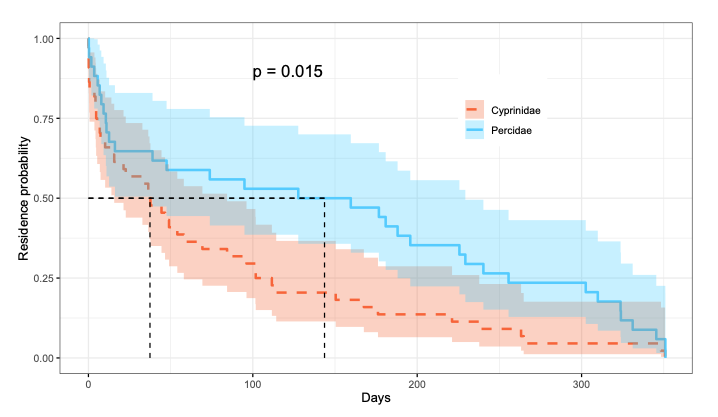
**


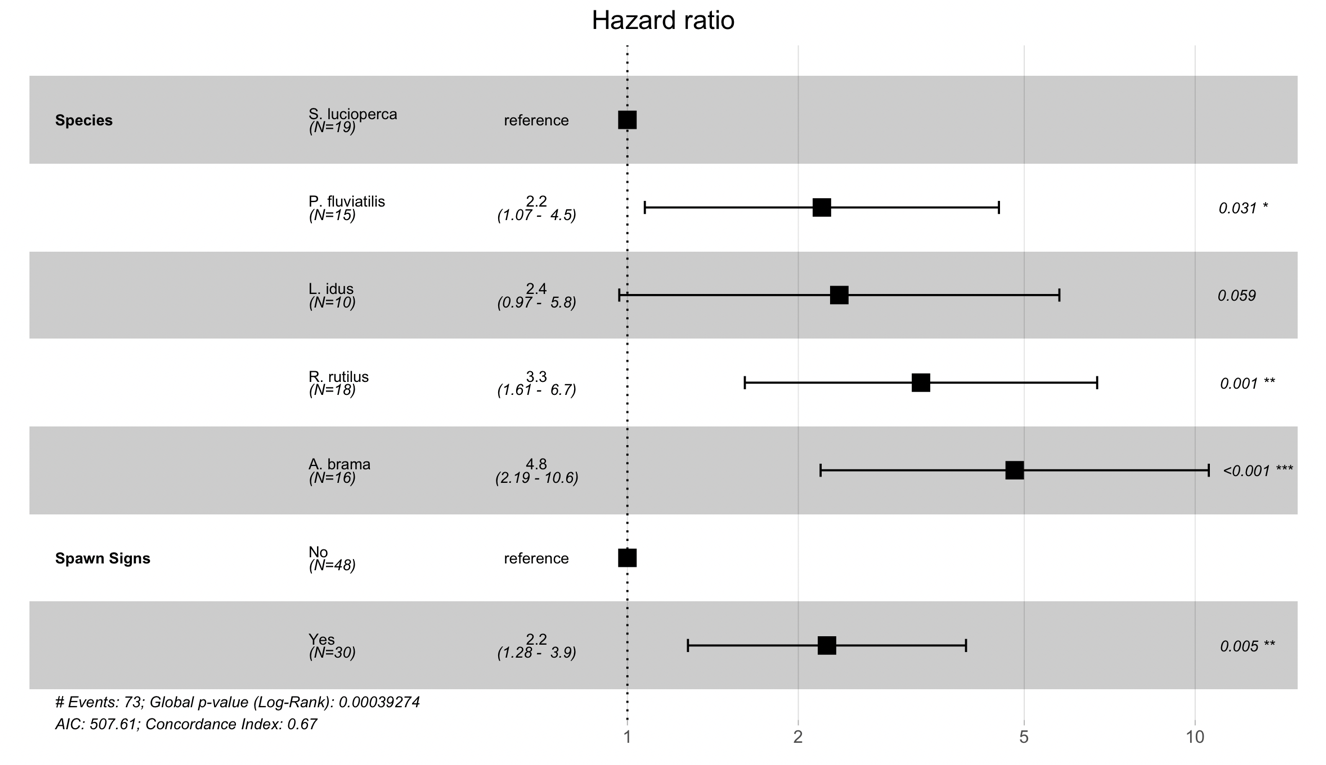
**Figure S1:** (**a**) Kaplan-Meijer survival curve displaying the number of days between the first and last detections of the five fish species per family in the study area (n = 78). The y-axis displays the portion of individuals still considered resident in the area. (**b**) A forest plot of a cox-proportional hazard analysis, performed at the species level and using the spawning likelihood of fish as a co-hazard. Significant deviation from the reference denotes an increased chance to leave the study area early.
